# LoG-staging: a rectal cancer staging method with LoG operator based on maximization of mutual information

**DOI:** 10.1186/s12880-025-01610-7

**Published:** 2025-03-06

**Authors:** Ge Zhang, Hao Dang, Qian Zuo, Zhen Tian

**Affiliations:** 1 School of Information Technology, Henan University of Chinese Medicine, 156 Jinshui Road, Zhengzhou, Henan 450046 China; 2 School of Medicine, Henan University of Chinese Medicine, 156 Jinshui Road, Zhengzhou, Henan 450046 China; 3 School of Computer Science and Artificial Intelligence, Zhengzhou University, 100 Science Avenue, Zhengzhou, Henan 450001 China

**Keywords:** Laplace of Gaussian (LoG) operator, MRI diagnostic image, Maximization of mutual information (MMI), Rectal cancer T-staging

## Abstract

Deep learning methods have been migrated to rectal cancer staging as a classification process based on magnetic resonance images (MRIs). Typical approaches suffer from the imperceptible variation of images from different stage. The data augmentation also introduces scale invariance and rotation consistency problems after converting MRIs to 2D visible images. Moreover, the correctly labeled images are inadequate since T-staging requires pathological examination for confirmation. It is difficult for classification model to characterize the distinguishable features with limited labeled data. In this article, Laplace of Gaussian (LoG) filter is used to enhance the texture details of converted MRIs and we propose a new method named LoG-staging to predict the T stages of rectal cancer patients. We first use the LoG operator to clarify the fuzzy boundaries of rectal cancer cell proliferation. Then, we propose a new feature clustering method by leveraging the maximization of mutual information (MMI) mechanism which jointly learns the parameters of a neural network and the cluster assignments of features. The assignments are used as labels for the next round of training, which compensate the inadequacy of labeled training data. Finally, we experimentally verify that the LoG-staging is more accurate than the nonlinear dimensionality reduction in predicting the T stages of rectal cancer. We innovatively implement information bottleneck (IB) method in T-staging of rectal cancer based on image classification and impressive results are obtained.

**Supplementary Information:**

The online version contains supplementary material available at 10.1186/s12880-025-01610-7.

## Introduction

As the third most common malignancy and the second most common cause of cancer-related death, colorectal cancer has always received considerable attention. The staging of rectal cancer through medical image screening plays an important role in treatment plan determination and neoadjuvant therapy [1]. The classification of converted magnetic resonance imagings (MRIs) from lesions in patients with rectal cancer based on convolution neural networks (CNN or ConvNet) model can discriminate the images into four categories, which is an important reference in determination of the subsequent treatment [2]. With the help of proper segmentation [3] and attention focus algorithm [4], ConvNets models achieve significant improvement in predicting the pathological T-staging with classification on MRIs. However, the effective features of classification only takes very small part in MRIs and the textures of carcinogenic tissue have fuzzy boundaries after data augmentation operation, which increase the difficulties in classification with deep learning models [5]. Moreover, the correctly labeled training data require a biospy after resection operation, which is the final judgment on the invasion site of rectal cancer. The complex operation also limits the amount of available training data which is a crucial factor in constructing effective classification models.

MRI technology accounts for the most commonly used diagnosis reference in rectal cancer [6], which generates millions of images every year in hospitals. The raw metadata contains significant potential in construction better classification models, but most of them remain unlabeled until pathological examination is applied. This calls for better classification models that can be trained on large amount of unlabeled data. Although the existing dataset can be augmented by operations like resize, crop, flip and rotate. The variance introduced in the augmentation can affect the model construction and in turn, affect the performance of classification. Moreover, it requires experienced radiologist to select the region of interest (ROI) due to anisotropy problem on medical images [7]. Some researchers also exploited features learned from non-medical domain to avoid the training drawbacks brought by inadequate training data [8, 9]. But the image variation between different stages is usually subtle imperceptible and the inadequacy in training directly affect the T categorization performance.

Unsupervised training of ConvNet is also utilized with clustering algorithms for image classification purpose [10]. The problem is that clustering algorithms are primarily designed for linear models on top of fixed features [11], which implies the data features are predetermined and constant, eliminating the requirement for a learning process to ascertain them. Taken *k*-means clustering as an example, training a ConvNet with this algorithm may collapse all the clusters into a single entity and lead to a trivial solution. Self-supervised learning is another popular form of unsupervised training [12]. It uses pretext tasks directly from the raw input data to replace human labeling. But these training methods need a well-designed pretext domain dependent task which is only feasible with proper expert knowledge. Recently, the image generation approach learns a mapping between predefined random noise and the images with a generative adversarial network (GAN) [13]. However, the two technologies above performs poorly on image classification with deep learning and very few literature are visible in application of medical image processing.

Aiming at solving the problems mentioned above, we propose a T-staging method based on Laplace of Gaussian (LoG) filter with maximization of mutual information (MMI) mechanism. First, LoG-staging filters the converted images with LoG operator to enhance texture details for better characterization in training. Then the filtered features are fed to the VGGNet [14] to alternate between descriptor clustering and cluster assignments prediction. The learned features are clustered with draw-and-merge strategy and used as additional labels in the training process of neural network. Finally, image features from MRIs are fully utilized to train an classification model, which can accurately predict the T stages of rectal cancer patients. LoG-staging provides an important reference for improving the treatment of rectal cancer in primary hospitals.

In summary, the contributions of this article can be summarized as follows:

- To facilitate the training of classification models with MRIs from rectal cancer, we propose a filtering method based on Laplacian of Gaussian operator to enhance the boundaries of tumors and pathological tissues.
- To utilize features from large amount of unlabeled training data, we propose a training method for image classification with MMI mechanism. The framework is robust to architecture modification and the distinguishable information in training dataset is preserved as much as possible.
- A rectal cancer staging method is constructed based on image classification model. The method is evaluated on clinical dataset and the discrimination accuracy on T2 and T3 is higher than human labeling by experienced radiologist.

The remainder of this article is organized as follows: Literature review section reviews the relevant literatures on medical image classification for T-staging purposes; Materials and methods section introduces the rectal cancer staging method based on LoG operator and MMI mechanism; Results section evaluates the performance of LoG-staging method. Finally, the experiment results are concluded in Conclusion section.

## Literature review

To cope with the inadequacy of labeled training data, several approaches train deep neural network in unsupervised manner. The most commonly seen approaches use *k*-means to pre-train ConvNets in a bottom-up manner to learn each layer sequentially [15]. But it is not an end-to-end training algorithm, which is suitable in learning features from images. There are a lot of similar clustering losses proposed for jointly learning ConvNet features and image clusters [16, 17]. However, they are not tested on any field to allow a deeper study on a specific task. Taken T staging application as an example, Jongin Kim et al. [18] propose a fully automatic discrimination of T2 and T3 rectal cancers using deep CNN, but they do not report the classification of images from T1 and T4 stages. Regarding to the distinguishable feature preservation concern, Bojanowski and Joulin [19] propose an exemplar support vector machine (SVM) to preserve information during visual features learning on a large dataset. But their method holds very high computation cost and they do not apply it to image classification tasks.

One possible solution to avoid the necessity of human labeling is to generate “pseduo-labels” directly from raw input data. Several domain independent approaches combine multiple cues to design a pretext task that lead to transferable features. They are recognized as “self-supervised learning” [20], which is a popular form of unsupervised learning that makes the best use of low-level information. Noroozi and Favaro [21] rearrange shuffled patches by training a network in spatial manner. Pathak et al. [22] guess the missing pixels based on their surrounding ones. An image retrial settings is also used to learn patch level convolutional kernel network [23]. Besides texture features in images, information contained in videos are also unignorable resource in computer vision tasks. Camera translation between consecutive frames are exploited through the temporal signal contained in tracked patches [24] and segmented videos [12]. Predefined random noise and training images are mapped in a parameterized manner with either an auto-encoder [25], a GAN [26] or reconstruction loss [27] for image generation. Apart from these approaches, image colorization and cross-channel prediction are also studied to preserve information better in training, especially application to T-staging of rectal cancer or any other tasks for medical image classification tasks [28, 29]. Our previous work [30] propose a vSLAM system based on LoG operator to deal with the variations in scale and rotation and we found this method is effective in medical image classification.

## Materials and methods

In this section, we first describe the MRI data conversion, filtering and augmentation details; then, we define the formal expressions used in the following algorithm and implementation of MMI mechanism; the clustering algorithm adopted in LoG-staging is described in the third section and finally the label generation is explained in detail.

**Fig. 1:**
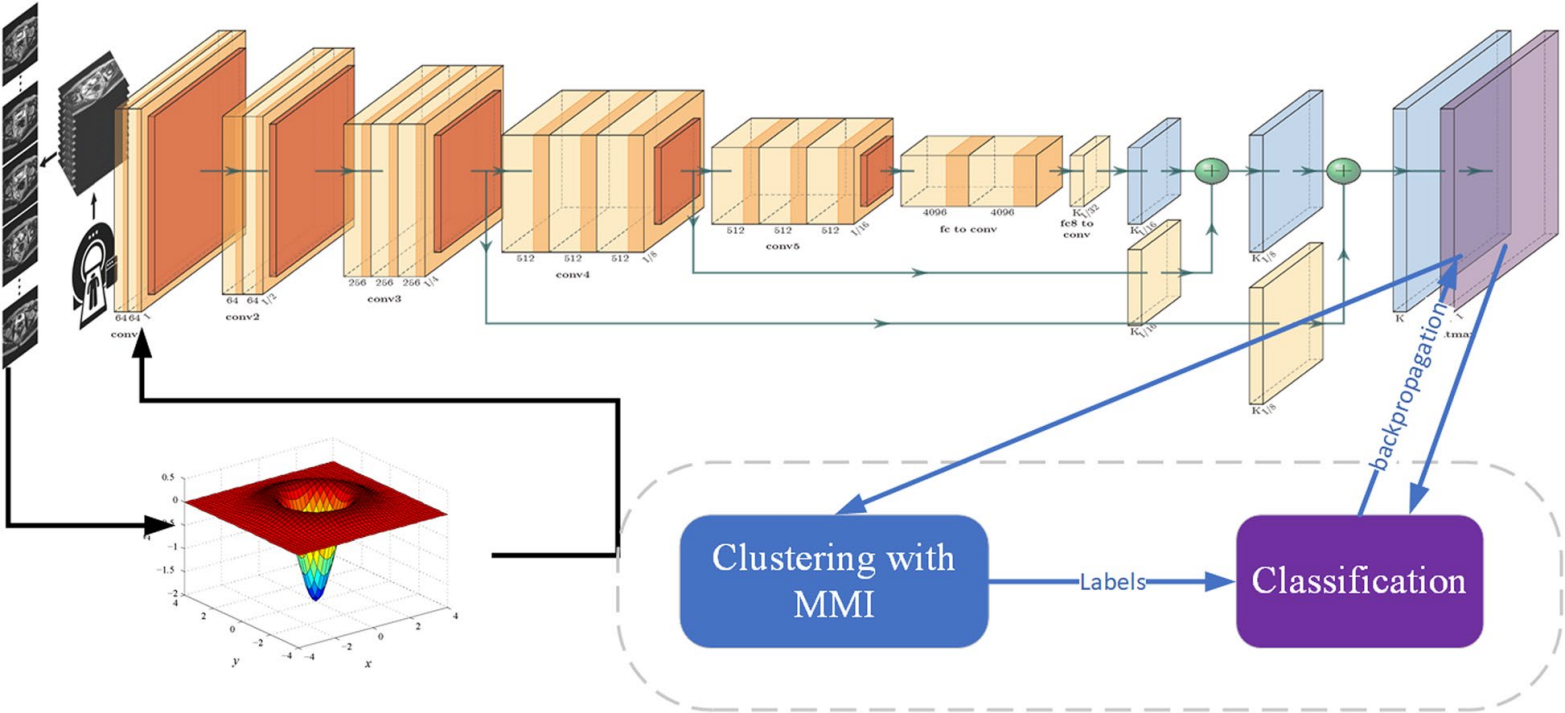
Flow chart of the LoG-staging method: we iteratively cluster deep features with MMI mechanism and use the cluster assignments as “labels” to learn the parameters of VGGNet

Specifically, the MRIs are converted to readable format after standard image registration and LoG filtering is applied to these converted images to enhance the texture details first, which is crucial in detecting the tiny variations of different stages. Then, an unsupervised training is applied to classification model with draw-and-merge strategy when cluster the output features of the VGGNet classification model and the subsequent cluster assignments are used as labels to optimize the lost function. Finally, we determine the T-staging category of the input rectal cancer MRIs based on the generated features thus providing constructive suggestions for T-staging diagnoses of clinical rectal cancer. The whole process is illustrated in Fig. 1.

### LoG filtering and data augmentation

The labeled MRI images are inadequate since the actual T-staging can only be determined after forensic medical examination. Training process needs large amount of labeled data and we use resize, crop, flip and rotate to augment the data after convert from 3D to 2D images. The augmentation operations on images bring variance and inconsistency in texture details, especially in regions with lesion boundaries. This can be the source of incorrect characterization and is recognized as anisotropy problem of volumetric images [31].

Specifically in MRIs of rectal cancer, conversion from 3D to 2D may result in fuzzy boundaries on lesion and organs. The obscure texture details remain a significant obstacle in characterization of learned features and training parameters of neural network. Regions with grayscale intensity variation in an image are typical sensitive areas in human eyes, which are the distinguishable features in images classification. The conversion process brings significant Gaussian noise and LoG operator is adopted to filter the noise to strengthen edges with obvious grayscale variation [30]. Experiment results demonstrate that LoG filtering is beneficial in discrimination of MRIs from different T stages.

The augmented images contain duplicate images in different scales and rotation states, which affect extracting distinguishable features negatively. According to our previous study in uncertainty on neural network [9], the scale invariance and rotation consistency are two crucial factors with great influence in data augmentation. Proper solutions on these problems are helpful in training on better classification models. LoG-staging adopts a four-level multi-scale image pyramid structured from 640 $\times$ 480 pixels to 80 $\times$ 60 pixels and Shi-Tomasi value thresholding [32] to reduce the affection of variations in scale and viewing angels. It is possible to character the distinguishable features of converted MRIs in a stable and continuous manner. Refer to literature [30] for more detailed description on the relevant operation.

The selection of Gaussian kernel is crucial in enhancing texture details of rectal cancer images. LoG-staging utilizes the following steps to determine the actual value of Gaussian kernel and implement filtering operation:

Suppose an 2D image is denoted as a function of *x* and *y*: $f(x*y)$. The Gaussian kernel is denoted as $G_{\sigma }(x,y)$, where *x* is the distance from the origin in the horizontal axis, *y* is the distance from the origin in the vertical axis, and $\sigma$ is the standard deviation of the Gaussian distribution. The Gaussian filtering process can be completed as a convolution operation between them.

According to the differentiation rules, the first derivative of Gaussian kernel is determined as

1 $$\begin{aligned} \frac{\partial }{\partial {x}}{{G}_{\sigma }}(x,y)=\frac{\partial }{\partial {x}}{{e}^{-}}^{({{x}^{2}}+{{y}^{2}})/2{{\sigma }^{2}}}=-\frac{x}{{{\sigma }^{2}}}{{e}^{-}}^{({{x}^{2}}+{{y}^{2}})/2{{\sigma }^{2}}} \end{aligned}$$

and the second derivative is determined as

2 $$\begin{aligned} \frac{{{\partial }^{2}}}{{{\partial }^{2}}x}{{G}_{\sigma }}(x,y) & =\frac{{{x}^{2}}}{{{\sigma }^{4}}}{{e}^{-}}^{({{x}^{2}}+{{y}^{2}})/2{{\sigma }^{2}}}\nonumber \\ & \quad -\frac{1}{{{\sigma }^{2}}}{{e}^{-}}^{({{x}^{2}}+{{y}^{2}})/2{{\sigma }^{2}}}=\frac{{{x}^{2}}-{{\sigma }^{2}}}{{{\sigma }^{4}}}{{e}^{-}}^{({{x}^{2}}+{{y}^{2}})/2{{\sigma }^{2}}}, \end{aligned}$$

The normalizing coefficient of Gaussian filter is omitted according to experience in [30]. Similar as the calculations above, we can get

3 $$\begin{aligned} \frac{{{\partial }^{2}}}{{{\partial }^{2}}y}{{G}_{\sigma }}(x,y)=\frac{{{y}^{2}}-{{\sigma }^{2}}}{{{\sigma }^{4}}}{{e}^{-}}^{({{x}^{2}}+{{y}^{2}})/2{{\sigma }^{2}}}, \end{aligned}$$

and the LoG operator is obtained by

4 $$\begin{aligned} LoG\triangleq \Delta {{G}_{\sigma }}(x,y)=\frac{{{x}^{2}}+{{y}^{2}}-2{{\sigma }^{2}}}{{{\sigma }^{4}}}{{e}^{-}}^{({{x}^{2}}+{{y}^{2}})/2{{\sigma }^{2}}}, \end{aligned}$$

where the second derivative of Gaussian kernel and convolution operation compose LoG operator. We can obtain kernels of any size by approximating the LoG expression above. Up to this point, Gaussian filtering on an image $f(x*y)$ can be obtained by the convolution between the Gaussian kernel and the image, which is formally expressed as:

5 $$\begin{aligned} \Delta [{{G}_{\sigma }}(x,y)*f(x,y)]=[\Delta {{G}_{\sigma }}(x,y)]*f(x,y) = LoG*f(x,y). \end{aligned}$$

The strong zero-crossings in the image are detected and kept to suppress the weak ones, which are likely caused by noise. Edges and details are strengthened and the boundaries become easier to distinguish in the feature extraction process. The scale invariance and rotation consistency are also improved with Laplacian sharpening, which is important in data augmentation.

The filter processed images have obvious extreme points in boundaries, which are regarded as edge response. LoG-staging needs to eliminate them since they affect the characterization of textures on edges and boundaries. We adopt the same way in [33] to find whether or not principal curvature is under a certain threshold. A feature point should be reserved for further usage when below the threshold, otherwise it should be discarded.

### Preliminaries and MMI

The preliminaries in this paper are given as follows: *N* filtered training images from patients with rectal cancer are denoted by $X=\{x_i|i=1,2,\cdots ,n\}$; Each one is annotated by a label $y_n \in \{0,1\}^k$, where *k* has four possible values 1,2,3 and 4, which correspond with four pathological stages; $\theta$ is the set of parameters learned during training and $f_\theta$ denotes the mapping in VGGNet model; The parameterized classifier $g_W$ predicts the stage that a single image comes from which is denoted by function $f_{\theta } (x_n)$.

The classification performance is closely related to its convolutional structure which gives a strong prior on the input data. LoG-staging tries to exploit the input data to bootstrap the discriminative capability of a VGGNet. The output of neural network is clustered via MMI mechanism and the subsequent assignments are used as labels to optimize the objective function which is defined as:

6 $$\begin{aligned} \min _{\theta , W}\frac{1}{N}\sum _{n=1}^{N}\mathcalligra{l}(g_W(f_\theta (x_n)), y_n), \end{aligned}$$

where $\mathcalligra{l}$ is the negative log-softmax function. This cost function is minimized using mini-batch stochastic gradient descent and back-propagation to compute the gradient [34]. The learned features $X^\prime$ can be recognized as representations of training images *X* and the labels are recognized as a set $Y=\{y_i|i=1,2,\cdots ,n\}$. LoG-staging seeks an optimal mapping of *X* into a more discriminative representation *T* such that the mutual information (MI) between *X* and *T* is minimized. On the contrary, the preservation of relevant information is maximized. Our goal is making most possible preservation of distinguishable texture details of MRIs. Specifically in classification of rectal cancer images, the compactness of classification representation is pre-defined as 4 stages and the preservation is the distinctive features contained in the images that identify the different stages. Maximization on the latter information result in more precise classification. This process can be mathematically expressed as:

7 $$\begin{aligned} \mathcal {L}_{max} = I(T;Y)-\lambda ^{-1}I(T;X), \end{aligned}$$

where $\lambda$ is the Lagrange multiplier to balance between data compression and relevant information preservation. Since the representation *T* is very small comparing with source information *X*, we concentrate on maximization of information preservation. According to our previous experience in [35], $\lambda$ is set as 100 in this process. LoG-staging implements the draw-and-merge strategy [36] to optimize Eq. 7.

### Draw-and-merge strategy

LoG-staging implements the draw-and-merge strategy to achieve MMI in the process of clustering the learned features $X^\prime$. Suppose all the images are captured from confirmed rectal cancer patients, so one image must belongs to one pathological stage of rectal cancer. LoG-staging implements sequential information bottleneck (sIB) [35] to optimize the objective function (7). First, $X^\prime$ from the training images are randomly partitioned into four clusters. Then, every individual element $x_n$ is sequentially “drawn” from the current cluster and recognized as a single cluster $\{x_n\}$. The quantity of clusters comes to five at this time. We have to “merge” $\{x_n\}$ into another cluster to guarantee that the number of clusters remains four. Let $\mathcal {L}^{bef}$ and $\mathcal {L}^{aft}$ denote the values of objective function before and after the merging process, respectively. $x_n$ has to be merged into a new cluster $t^{new}$, which satisfies $t^{new}=argmin(\mathcal {L}^{bef}-\mathcal {L}^{aft})$ to maximize the objective function. Element $x_n$ that was drawn from the current cluster is merged into another cluster to minimize the information loss of the objective function in clustering with draw-and-merge strategy. We refer to the difference between values of objective function before and after the merger as “merger cost”, which is expressed as:

8 $$\begin{aligned} \Delta \mathcal {L} & = \mathcal {L}^{bef}-\mathcal {L}^{aft}\nonumber \\ & =(I(T^{bef};Y)-I(T^{aft};Y))\nonumber \\ & \quad -\lambda ^{-1}(I(T^{bef};X)-I(T^{aft};X))\nonumber \\ & \equiv \Delta {{I}_{2}}-{{\lambda }^{-1}}\Delta {{I}_{1}}. \end{aligned}$$

According to the definitions and proofs in [37], we have

9 $$\begin{aligned} \Delta {{I}_{2}}=p(\bar{t})\centerdot JS_{\prod }[p(x),p(y|t)], \end{aligned}$$

10 $$\begin{aligned} \Delta {{I}_{1}}=p(\bar{t})\centerdot JS_{\prod }[p(x),p(x|t)], \end{aligned}$$

where $JS_{\prod }$ denotes the Jensen-Shannon divergence and $\bar{t}$ is the new cluster which $\{x_n\}$ merged to. We calculate the merger cost in every assignment to find the particular $\bar{t}$ that Eq. 7 is optimized. The assignment with minimum merger cost is chosen and $\{x_n\}$ is merged to it. The draw-and-merge converges when every drawn $x_n$ is merged to its current cluster. In that case, the clustering algorithm converges to a stable status and the inter-cluster difference is maximized. Refer to [35] for more detailed description on the optimization.

Benefiting from the optimization process described above, the distinctive texture features that identify four stages of rectal cancer are preserved as much as possible to classify the images into four categories. LoG-staging will find a distinctive clustering result that distinguish the four stages to the best and the result is feed to VGGNet model for another round of training. Since the unlabeled data is large enough, we can collect enough “labels” in this way for a better training result. The model will “learn” enough distinctive texture features to classify potential rectal cancer images into four categories, which corresponds to four pathological stages.

### Algorithm construction

LoG-staging converts volumetric image into 2D and filters them to enhance the local contrast, which improve the identification of textures simultaneously. Augmentation operations, e.g. flip, crop and rotate, are performed on the filtered images and MMI clustering clusters the central cropped images features as four distinctive clusters. The cluster labels are recognized as the stage labels for rectal cancer and fed into the training process as labeled data. Since the clustering utilizes “draw-and-merge” strategy, the objective function is maximized and the information loss is minimized in this process. In other words, the distinctive texture features that recognize rectal cancer stages are preserved as much as possible. LoG-staging trains a better classification model to categorize the converted images. The label generation process is summarized as Algorithm 1.

**Figure:**
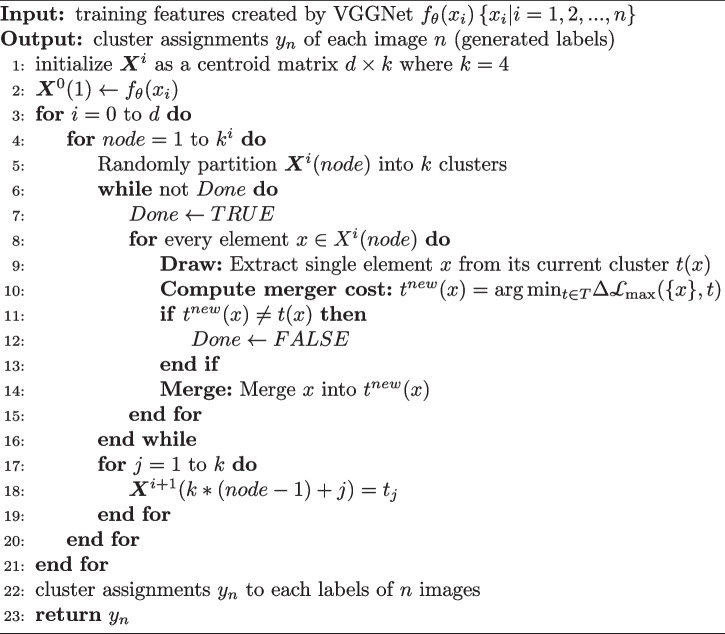
**Algorithm 1** The label generation process of LoG-staging method

A set of optimal assignments $y^*_n$ and a centroid matrix $\varvec{X}^*$ are generated in the clustering process and the assignments are used as generated labels to optimize cost function (6). LoG-staging iteratively learns the features and clusters them as generated labels with draw-and-merge strategy, compensating the inadequate labeled MRIs for better classification model.

### Complexity analysis

The label generation process mainly consists of the implementation of sIB, which has a higher time complexity than traditional cluster algorithm. We need to compute the merger costs with respect to each cluster *T* in every draw-and-merge operation, which has a time complexity on the order of *O*(|*T*||*Y*|). Thus, the computation complexity of label generation algorithm is bounded by *O*(*k*|*X*||*T*||*Y*|), where *k* denotes the number of classes. There are four values of rectal cancer T stages: T1, T2, T3 and T4 which correspond to the four classes of unlabeled data. Although the iteration of “draw-and-merge” can not be determined until algorithm reaches a convergent status, the run-time of LoG-staging is acceptable for our current application because $k\cdot |T|\ll |X|^2$ in most cases.

## Results

In this section, we carry out experiments to validate the improvement of our method with LoG operator in terms of area-under-curve (AUC), accuracy, sensitivity, and specificity, by means of contrast relationship between accuracy and recall against the state-of-the-art T-staging methods. We also carry out separate ablation study on the affection of LoG filtering and MMI mechanism, which are two main contributions of LoG-staging. Additionally, the architecture robustness of LoG-staging is evaluated by substitute VGGNet by AlexNet and ResNet. To demonstrate the effectiveness and value of LoG-staging in clinical practice, we compare the performance of our method and other deep learning based methods with staging annotations labeled by experienced radiologists.

### Baseline characteristics

**Fig. 2:**
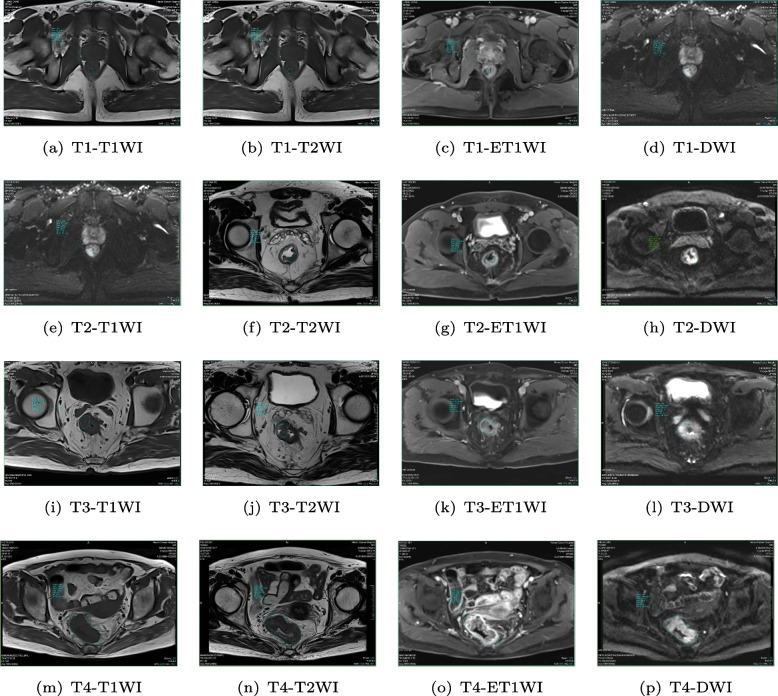
Illustrations of T1WI, T2WI, ET1WI and DWI images of rectal cancer MRIs from pathological stages T1 to T4. The T2-weighted image (T2WI) is routinely used to assessing the pathological T stages of rectal cancer [38], LoG-staging incorporates T1-weighted image (T1WI) in inverse phase and ET1WI for better characterization of lesion boundaries. Diffusion weighted imaging (DWI) (b values of 0 and $1000 s/mm^2$) is also obtained in the transverse section. MRIs in different modalities guarantee complete characterization of texture details in the training process

We collected data from 729 rectal cancer patients from the First Affiliated Hospital of Henan University of Chinese Medicine, and each image is annotated by radiologist after examination with colonoscope. Due to current limitations of MRI technology, it is difficult to accurately determine the T-staging of rectal cancer with a single inspection method. Four different methods are typically used to comprehensively determine the T-staging of rectal cancer patients: T1WI, T2WI, ET1WI and DWI. The illustrations of converted images from the four stages are shown in Fig. 2.

The proportion of rectal cancer patients in the T1, T2, T3, and T4 stages was 14%, 29%, 43%, and 18%, respectively. After the data of 331 patients who did not meet the experimental criteria were excluded, the proportion of rectal cancer patients in the different stages was 18%, 27%, 35%, and 20%. For each T stage, MRI T1 weighted imaging (T1WI), T2 weighted imaging (T2WI), enhanced T1WI (ET2WI) and diffusion weighted imaging (DWI) techniques [39] were used to determine the clinical rectal cancer imaging diagnosis. We use 10-fold cross validation to verify the generalization ability of LoG-staging algorithm in performance comparison and unlabeled images as cluster dataset in ablation study. The patient characteristics are summarized in Table 1, where $\times$ indicates that there is no record of this data type in the rectal cancer diagnostic MRI images, and 0 indicates that the tumor is not displayed in the image.

**Table 1:** Baseline characteristics of patients

Variables	T1-staging	T2-staging	T3-staging	T4-staging
Age (years)	53.5±7.5	$58.5\pm 14.5$	$60.2\pm 21.8$	$59.6\pm 5.4$
Sex (male)	51	64	83	43
Distance positioning (cm)	3±2	6±3.5	8.5±2.5	12.5±2.5
Massive (Longitudinal diameter, mm)	$\times$	25.3±7.7	34.5±8.5	$\times$
Intestinal wall infiltration (Longitudinal diameter, mm)	18.7±5.3	49.2±7.8	35.9±15.1	40.4±12.6
The circumference of the lesion surrounding the bowel	$<1/4$	$1/<span class='convertEndash'>4-1</span>$	$1/<span class='convertEndash'>2-1</span>$	$3/<span class='convertEndash'>4-1</span>$
Tumor infiltration size (mm)	0	0	8.5±7.8	$>15$
Training cohort	50	75	98	56
Validation cohort	22	32	41	24
Number of patients	72	107	139	80
Ratio of T-staging patients (%)	18	27	35	20

### Performance comparison of classification model

We compare LoG-staging with the state-of-the-art classification models: Simple CNN [40], AlexNet [41] and Inception [42]. The classification performance on the four stages are evaluated by accuracy, sensitivity, specificity and balanced accuracy. The accuracy index is computed as:

11 $$\begin{aligned} Accuracy = \frac{TP+TN}{TP+TN+FP+FN} \end{aligned}$$

where TP, TN, FP and FN refers to true positive, true negative, false positive and false negative results, respectively. The sensitivity is a measure on how sensitive is the algorithm on detection on different stages which is computed as:

12 $$\begin{aligned} & Sensitivity (P,T) = \frac{|P_0\cap T_0|}{T_0}, \nonumber \\ & Specificity (P,T) = \frac{|P_1\cap T_1|}{T_1} \end{aligned}$$

where $P_0$ and $P_1$ represents a particular pixel predicted as negative and positive for a particular stage and $T_0$ and $T_1$ represents the ground truth labeled as negative and positive pixel. The balanced accuracy is the average of specificity and sensitivity.

Table 2 shows the classification performance of LoG-staging and its competitors (Simple CNN, AlexNet and Inception). The results show our method achieves higher accuracy and lower specificity in discrimination on T stages than other comparative approaches, especially in T2 and T3 stages. Simple CNN has a 0 value on sensitivity in all four stages discrimination. AlexNet achieves higher accuracy and sensitivity in classification on images with rectal cancer. The accuracy of discrimination goes beyond 0.8 in the classification with Inception model, which reaches 0.83 in T2 and 0.84 in T3. Our method achieves the highest accuracy in all four models, which accounts 0.82 and 0.85 in T2 and T3 stages respectively. Although the sensitivity is slightly lower than Inception, LoG-staging detects 95% positives results in classification of rectum images from T2 stage and 87% positive ones from T3 stage. The model achieves a mean accuracy of 0.82, sensitivity of 0.91 and nearly 1 of specificity. The enhanced edges and details facilitates classification models to recognized the tumor boundaries and in turn, improves the staging performance based on the feature extraction of MRI images.

**Table 2:** Performance comparison of classification model evaluated on test set

Algorithm	T1-staging				T2-staging				T3-staging				T4-staging			
	Acc.	Sen.	Spec.	Bac.	Acc.	Sen.	Spec.	Bac.	Acc.	Sen.	Spec.	Bac.	Acc.	Sen.	Spec.	Bac.
Simple CNN	0.43	0	1	0.5	0.45	0	1	0.5	0.46	0	1	0.5	0.42	0	1	0.5
AlexNet	0.76	0.58	1	0.79	0.74	0.61	1	0.81	0.79	0.63	1	0.82	0.71	0.63	1	0.81
Inception	0.82	0.94	0.74	0.84	0.83	0.90	0.76	0.83	0.84	0.84	0.78	0.81	0.66	0.97	0.9	0.94
LoG-staging	0.84	0.91	1	0.96	0.82	0.95	0.93	0.94	0.85	0.87	0.96	0.92	0.74	0.91	1	0.96

### Ablation study

LoG-staging differs from traditional classification models in two sections: LoG filtering and MMI mechanism. To see the efficiency of the two parts, we perform an ablation study in this part. First, we remove LoG filtering before feeding the extracted features to training process to verify the effectiveness of edge enhancement. Second, we replace “draw-and-merge” strategy clustering with *k*-means to test the effectiveness of MMI mechanism. For each separated test, the ROC curve is plotted in accordance with T stages to demonstrate the affection in classification accuracy in each stage.

**Fig. 3:**
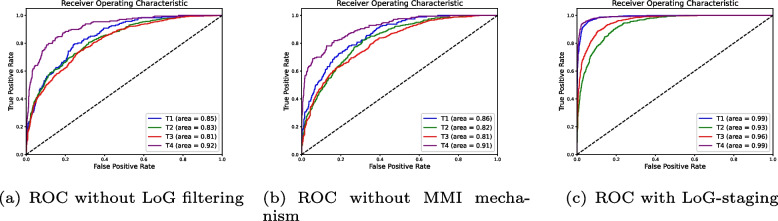
Comparison of ROC curves among the four classification of stages without LoG filtering and MMI mechanism

The significance of LoG filtering is obvious when referring to the comparison of TPR (true positive rate) and FPR (false positive rate) as illustrated in Fig. 3. We can see the curves tend to cover smaller area when LoG filtering and MMI clustering are removed from the classification. The detection of MRI images from T1 stage keeps the best in accuracy when removing the two steps, which only drops by 0.14 and 0.13 in coverage in ROC without LoG filtering and ROC with *k*-means, respectively. One possible explanation is that the invasion level is shallow and the texture details are more obvious. When it comes to the classification of images from T2 and T3 stages, the accuracy and recall drops by 0.2 and 0.3 since the original texture details can hardly tell the difference between the invasion site of the two stages. Our improvement can help the classification model to distinguish between the two stages by providing more obvious boundaries and textures. In the classification of MRI images from T4 stage, the affection is not that obvious comparing with T2 and T3 because the images already holds a clear difference comparing with the other three stages. LoG-staging can improve the detection and classification of images from T2 and T3 stages, which are more clinically valuable comparing with T1 and T4.

Performance comparisons are also carried out on the competitors to validate the effectiveness of LoG filtering and generated labels with self-supervised learning. To test the robustness on architecture modification, we implement ResNet and AlexNet as substitute for VGGNet in classification model construction. For simplicity purpose, we only report the average value of accuracy, sensitivity and specificity of LoG-staging and its competitors.

Table 3 contains two part separated with a horizontal line. The upper part collects the performance details on different methods and the lower part collects data obtained with different network architectures. There is a decrease in accuracy by 14% with removal of LoG filtering, which suggests the importance of edges characterization in classification. The table suggests that advantage of unlabeled data only remains in the field of sensitivity. When the clustering algorithm is replaced with *k*-means without “draw-and-merge” mechanism, accuracy is slightly lower than two competitors’ (AlexNet 1% and Inception 5%). But the sensitivity performance is acceptable. When it comes to robustness evaluation, architectures like VGGNet or ResNet have a much higher classification accuracy on ImageNet than network like AlexNet in supervised settings and we should expect the same result in unsupervised training. We test the three network architectures in rectal cancer image classification with LoG-staging. The accuracy remains at the same level with VGGNet and ResNet, which means our method has robust performance with deeper network like VGGNet and ResNet. It also suggests that deeper network leads to significant improvement in classification task.

**Table 3:** Accuracy, sensitivity, specificity of classification model and robustness comparison on network architectural modifications

Algorithm	Acc.	Sen.	Spec.	Bac.
Simple CNN	0.44	0	1	0.5
AlexNet	0.75	0.61	1	0.8
Inception	0.79	0.91	0.79	0.85
LoG-staging without LoG filtering	0.67	0.83	1	0.92
LoG-staging without MMI mechanism	0.74	0.88	1	0.94
LoG-staging(VGGNet)	0.81	0.91	0.97	0.94
LoG-staging(ResNet)	0.82	0.89	0.98	0.94
LoG-staging(AlexNet)	0.73	0.63	0.96	0.8

### Performance evaluation with radiologist

The discrimination results above are evaluated based on the ground truth label through bio-assay assessment after interventional treatment. We are also interested in the performance comparison with professional radiologist with MRIs only. Two studies have reported the staging result based on MRI only by radiologists in classify T2 and T3 stages of rectal cancer. According to the clinical practice, we usually category T1 and T2 as a single stage and ignore the results of T4 since the features are obvious with visual recognition. So the performance comparison is carried out only on accuracy and recall of T2 and T3 stages.

Table 4 compares the staging information created by professional radiologists and deep learning based algorithms. Although the dataset used are different and direct comparison is impossible, we can still see how competitive LoG-staging is. The accuracy accounts nearly 20% higher than human being and 2% higher than 3D model. The recall rate is far better than 3D model in T2 stage and slightly lower in T3. Because the T1 and T2 are usually combined in clinical practice, radiologists achieve higher recall rate in classification of images from the two stages. However, the recall rate of classification on T3 images is significantly lower than deep learning models, which demonstrates the superiority of unsupervised learning labels in image classification. LoG-staging ignores the depth information of images in the conversion from 3D into 2D modality and we believe there is still room for improvement if this information is taken into consideration.

**Table 4:** Classification performance of T2/T3 stages of rectal cancer by radiologists, the competitor and LoG-staging

Method	AUC	Acc	Recall(T2-staging)	Recall(T3-staging)
Ang et al. [43]	-	68.87	85.5	50.1
Maas et al. (1.5T MR) [44]	0.73	66.67	83.33	52.38
Maas et al. (3.0T MR) [44]	0.64	56.41	72.22	42.86
$rMC_5$+Bilinear+Triplet [45]	0.831	79.3	62.1	86.7
LoG-Staging	0.876	81.4	73.34	79.8

## Conclusion

This paper proposes a staging method named LoG-staging based on image classification to preoperative discriminate the four pathological stages of rectal cancer from MRI images. LoG-staging consists of two parts: the LoG filtering process to enhance the texture details of gray-scale image for better invariance, which is crucial in data augmentation; and a self-supervised training method for image classification with MMI mechanism. This training method enables label generation operation by clustering the training output, which is useful in utilizing the unlabeled training data. Moreover, LoG-staging clusters the features with MMI mechanism which performs well in extracting the most distinctive information in constructing classification models. The useful information is preserved as much as possible and redundant details are reduced in this process. We compare the performance of LoG-staging with three competitors who have the same discrimination mechanism with ours and staging prediction from experience radiologists. Moreover, we also carried out an ablation study to investigate the contribution of LoG filtering and clustering with MMI mechanism. The experiment results suggest that LoG-staging performs better in discriminate between T2 and T3 stages and holds higher recall rate comparing with limited manually labeled MRIs only as training data. The sensitivity and specification are also higher in the classification of rectal cancer images, especially in images from T2 and T3 stages which have smaller variance in texture details. But LoG-staging is far from perfect, we do not make use of the depth information of MRIs since the 3D images are converted to 2D for filtering operation. We should be targeting at performing classification on 3D images directly. The computed tomography (CT) still remains as the main diagnostic modality for initial staging of rectal cancer and we will study the possibility of utilization.

## Supplementary Information

Supplementary Material 1.

## Data Availability

The data presented in this study are available on request from the corresponding author due to ethical reasons.

## Code availability

The source of LoG-staging will be made available on public repository after the paper is accept for publication.
