# Supplementary material for: LoG-staging: a rectal cancer staging method with LoG operator based on maximization of mutual information
Source: BMC Med Imaging. 2025 Mar 6;25:78. doi: 10.1186/s12880-025-01610-7 (PMC11887235; doi:10.1186/s12880-025-01610-7)
Supplement: Supplementary file 1 — Supplementary Material 1. [file 12880_2025_1610_MOESM1_ESM.zip › T21-eps-converted-to.pdf]

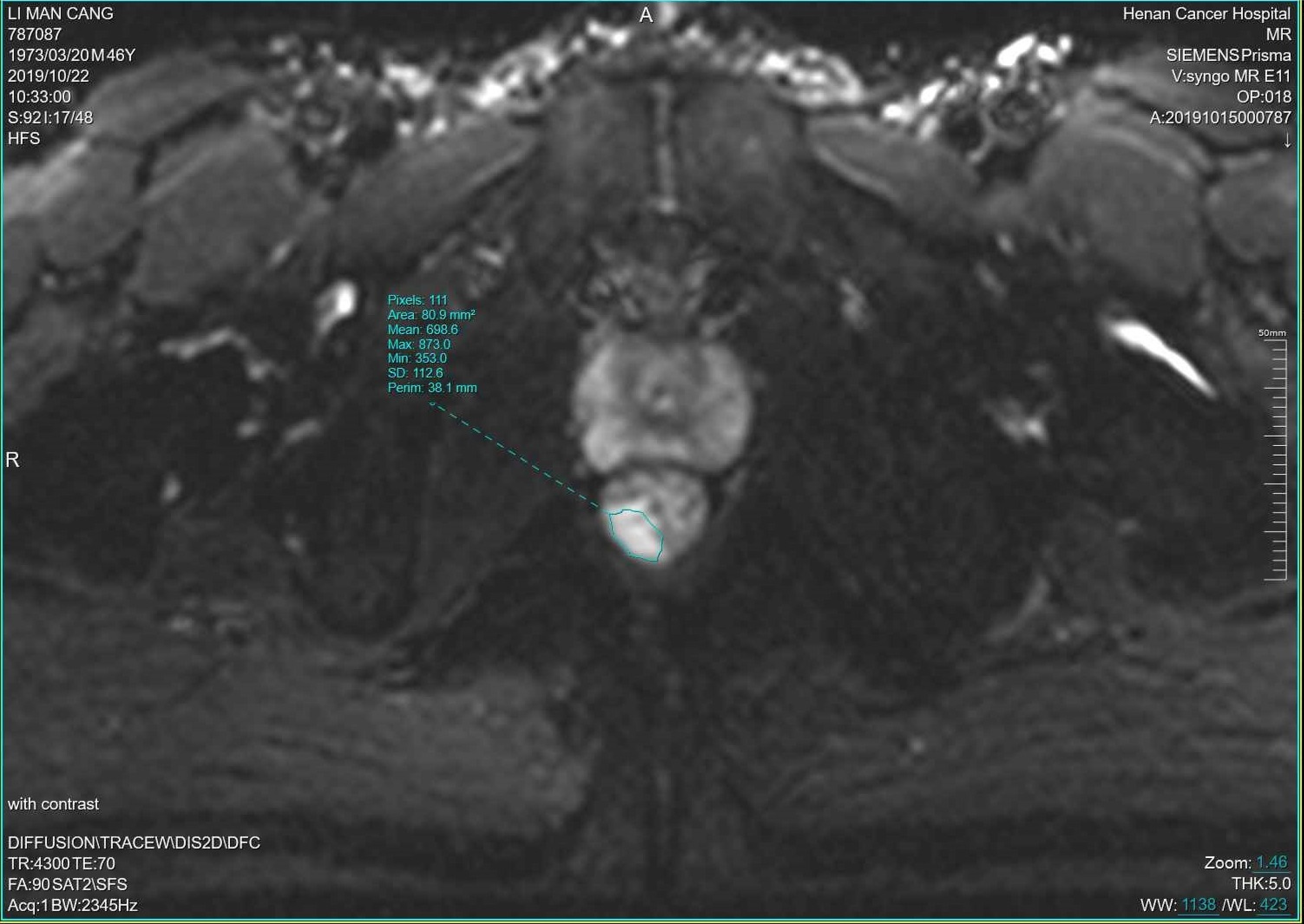
LI MAN CANG
787087
1973/03/20 M 46Y
2019/10/22
10:33:00
S:92 I:17/48
HFS
A
Henan Cancer Hospital
MR
SIEMENS Prisma
V:syngo MR E11
OP:018
A:20191015000787
Pixels: 111
Area: 80.9 mm²
Mean: 698.6
Max: 873.0
Min: 353.0
SD: 112.6
Perim: 38.1 mm
R
50mm
with contrast
DIFFUSION\TRACEW\DIS2D\DFC
TR:4300 TE:70
FA:90 SAT2\SFS
Acq:1 BW:2345Hz
Zoom: 1.46
THK:5.0
WW: 1138 /WL: 423
