# Supplementary material for: LoG-staging: a rectal cancer staging method with LoG operator based on maximization of mutual information
Source: BMC Med Imaging. 2025 Mar 6;25:78. doi: 10.1186/s12880-025-01610-7 (PMC11887235; doi:10.1186/s12880-025-01610-7)
Supplement: Supplementary file 1 — Supplementary Material 1. [file 12880_2025_1610_MOESM1_ESM.zip › T31-eps-converted-to.pdf]

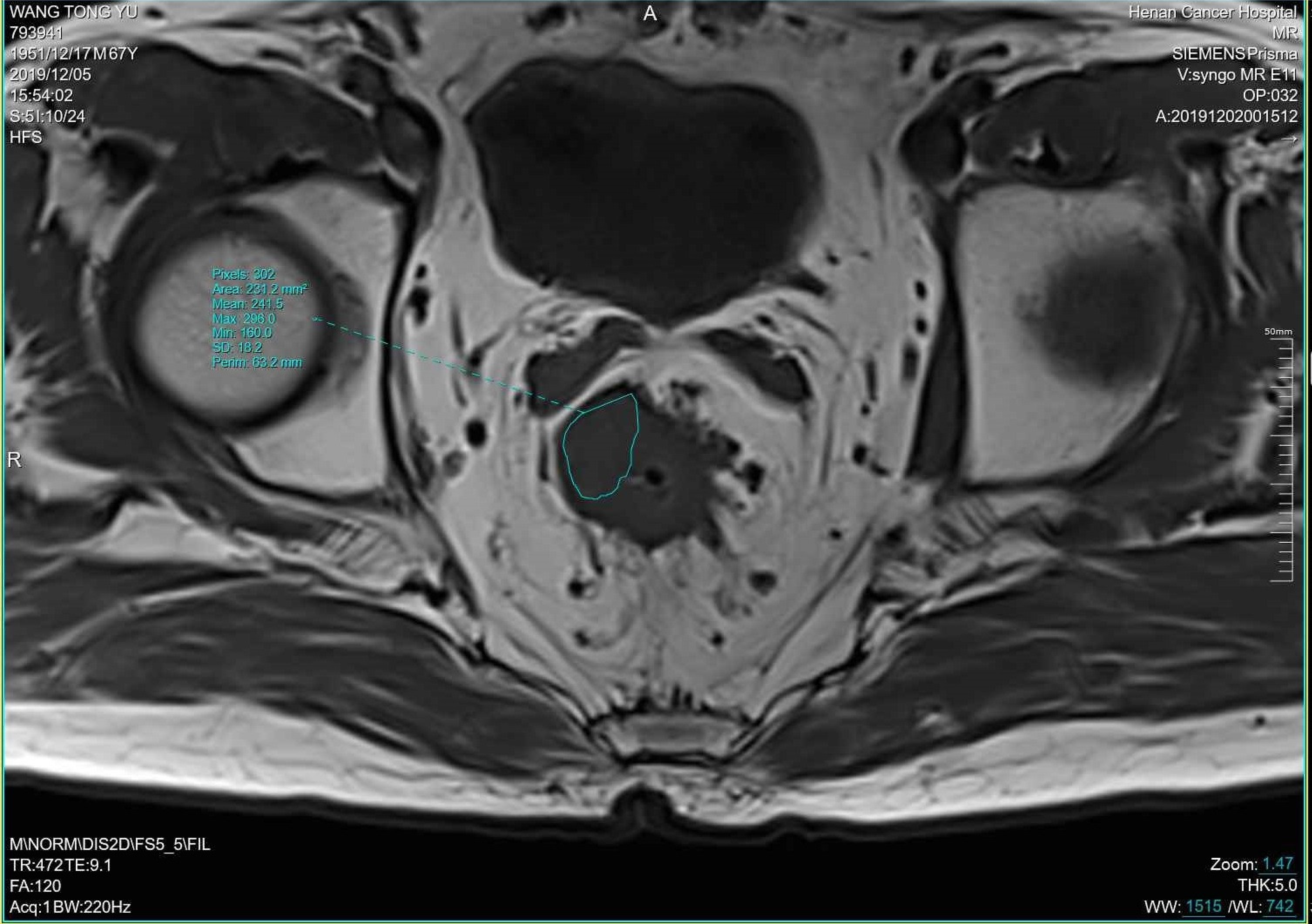
WANG TONG YU
793941
1951/12/17 M 67Y
2019/12/05
15:54:02
S:5 I:10/24
HFS
A
Henan Cancer Hospital
MR
SIEMENS Prisma
V:syngo MR E11
OP:032
A:20191202001512
Pixels: 302
Area: 231.2 mm²
Mean: 241.5
Max: 296.0
Min: 160.0
SD: 18.2
Perim: 63.2 mm
50mm
R
M\NORM\DIS2D\FS5_5\FIL
TR:472 TE:9.1
FA:120
Acq:1 BW:220Hz
Zoom: 1.47
THK:5.0
WW: 1515 /WL: 742
