# Supplementary material for: LoG-staging: a rectal cancer staging method with LoG operator based on maximization of mutual information
Source: BMC Med Imaging. 2025 Mar 6;25:78. doi: 10.1186/s12880-025-01610-7 (PMC11887235; doi:10.1186/s12880-025-01610-7)
Supplement: Supplementary file 1 — Supplementary Material 1. [file 12880_2025_1610_MOESM1_ESM.zip › T32-eps-converted-to.pdf]

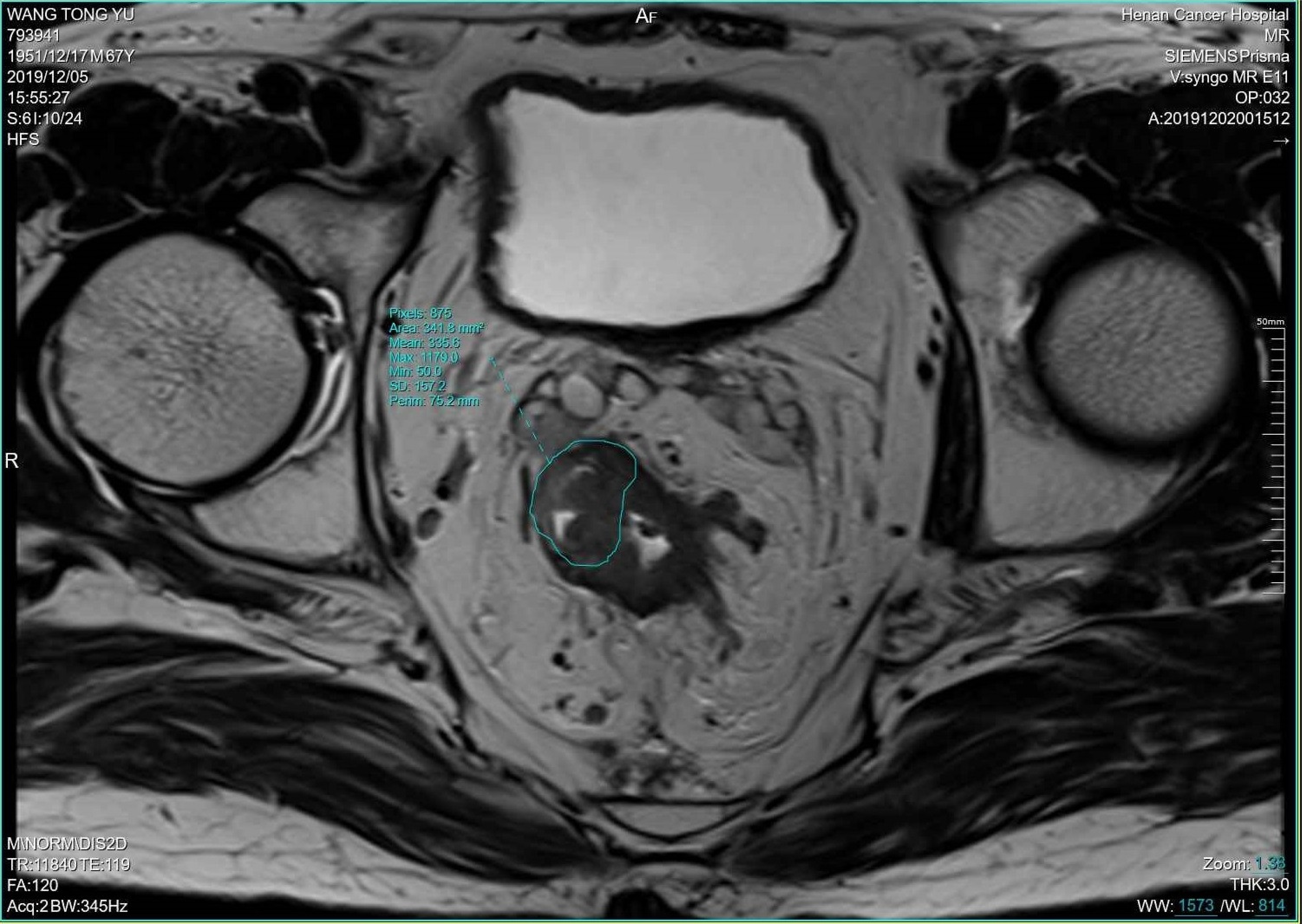
WANG TONG YU
793941
1951/12/17 M 67Y
2019/12/05
15:55:27
S:6 I:10/24
HFS
AF
Henan Cancer Hospital
MR
SIEMENS Prisma
V:syngo MR E11
OP:032
A:20191202001512
Pixels: 875
Area: 341.8 mm²
Mean: 335.6
Max: 1179.0
Min: 50.0
SD: 157.2
Perim: 75.2 mm
50mm
R
M\NORM\DIS2D
TR:11840 TE:119
FA:120
Acq:2 BW:345Hz
Zoom: 1.38
THK:3.0
WW: 1573 /WL: 814
