# Supplementary material for: LoG-staging: a rectal cancer staging method with LoG operator based on maximization of mutual information
Source: BMC Med Imaging. 2025 Mar 6;25:78. doi: 10.1186/s12880-025-01610-7 (PMC11887235; doi:10.1186/s12880-025-01610-7)
Supplement: Supplementary file 1 — Supplementary Material 1. [file 12880_2025_1610_MOESM1_ESM.zip › T34-eps-converted-to.pdf]

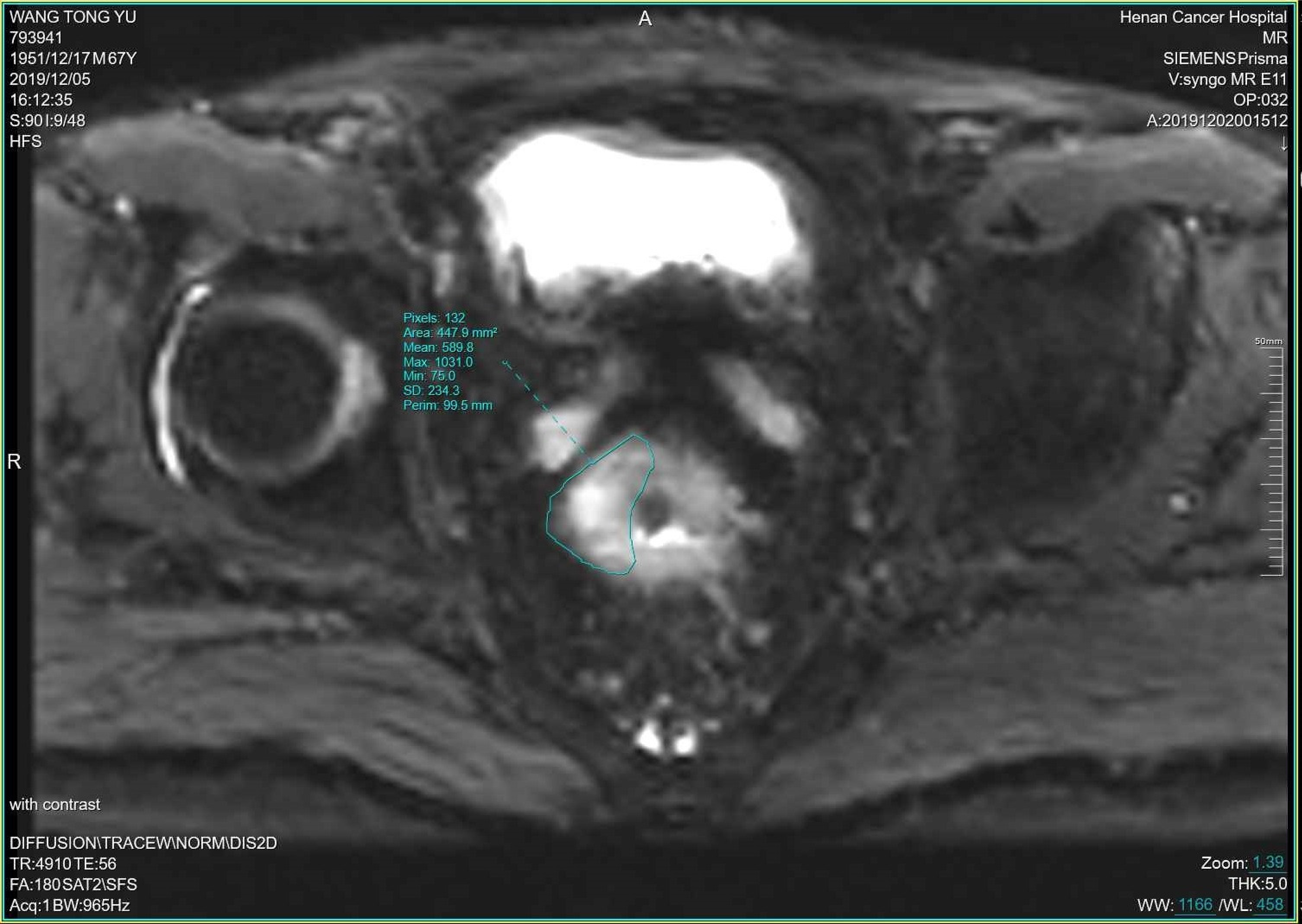
WANG TONG YU
793941
1951/12/17 M 67Y
2019/12/05
16:12:35
S:90 I:9/48
HFS
A
Henan Cancer Hospital
MR
SIEMENS Prisma
V:syngo MR E11
OP:032
A:20191202001512
Pixels: 132
Area: 447.9 mm²
Mean: 589.8
Max: 1031.0
Min: 75.0
SD: 234.3
Perim: 99.5 mm
50mm
R
with contrast
DIFFUSION\TRACEW\NORM\DIS2D
TR:4910 TE:56
FA:180 SAT2\SFS
Acq:1 BW:965Hz
Zoom: 1.39
THK:5.0
WW: 1166 /WL: 458
