# Supplementary material for: LoG-staging: a rectal cancer staging method with LoG operator based on maximization of mutual information
Source: BMC Med Imaging. 2025 Mar 6;25:78. doi: 10.1186/s12880-025-01610-7 (PMC11887235; doi:10.1186/s12880-025-01610-7)
Supplement: Supplementary file 1 — Supplementary Material 1. [file 12880_2025_1610_MOESM1_ESM.zip › T22-eps-converted-to.pdf]

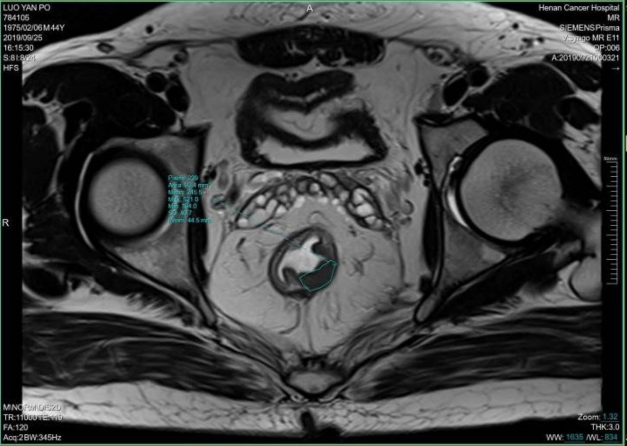

LUO YAN PO
784105
1975/02/06 M 44Y
2019/09/25
16:15:30
HFS
A
R
Henan Cancer Hospital
MR
SIEMENS Prisma
OP:006
A:20190921000321
50mm
Zoom: 1.32
THK:3.0
WW: 1635 /WL: 834
TR:11000 TE:119
FA:120
Acq:2 BW:345Hz
