# Supplementary material for: LoG-staging: a rectal cancer staging method with LoG operator based on maximization of mutual information
Source: BMC Med Imaging. 2025 Mar 6;25:78. doi: 10.1186/s12880-025-01610-7 (PMC11887235; doi:10.1186/s12880-025-01610-7)
Supplement: Supplementary file 1 — Supplementary Material 1. [file 12880_2025_1610_MOESM1_ESM.zip › T23-eps-converted-to.pdf]

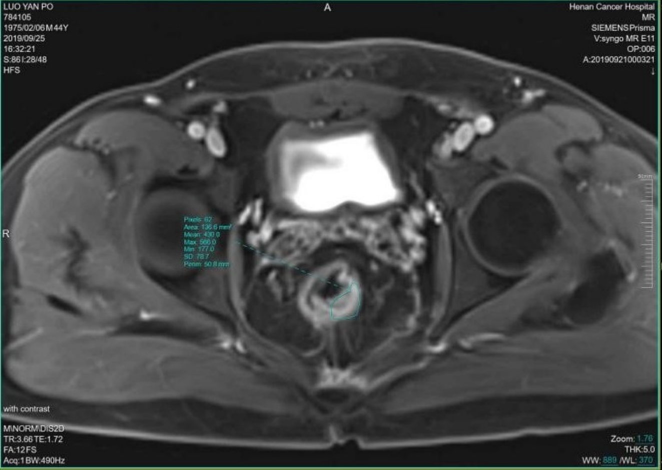
LUO YAN PO
784105
1975/02/06 M 44Y
2019/09/25
16:32:21
S:86 I:28/48
HFS
A
Henan Cancer Hospital
MR
SIEMENS Prisma
V:syngo MR E11
OP:006
A:20190921000321
R
Pixels: 62
Area: 136.6 mm²
Mean: 430.0
Min: 177.0
SD: 78.7
Perim: 50.8 mm
with contrast
M\NORM\DIS2D
TR:3.66 TE:1.72
FA:12 FS
Acq:1 BW:490Hz
Zoom: 1.76
THK:5.0
WW: 889 /WL: 370
