# Supplementary material for: LoG-staging: a rectal cancer staging method with LoG operator based on maximization of mutual information
Source: BMC Med Imaging. 2025 Mar 6;25:78. doi: 10.1186/s12880-025-01610-7 (PMC11887235; doi:10.1186/s12880-025-01610-7)
Supplement: Supplementary file 1 — Supplementary Material 1. [file 12880_2025_1610_MOESM1_ESM.zip › T24-eps-converted-to.pdf]

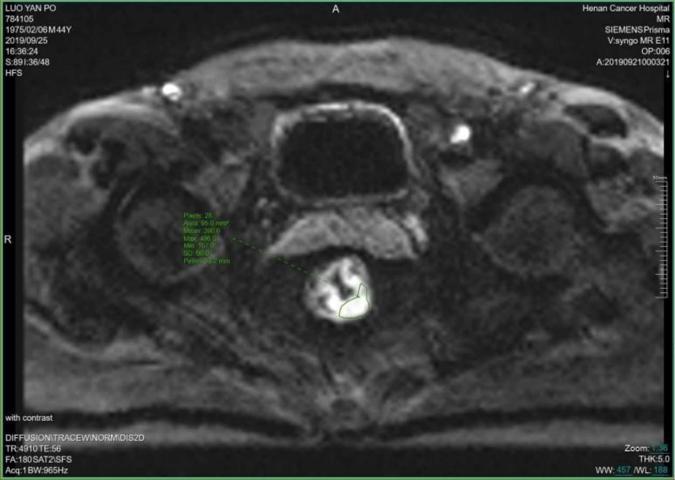
LUO YAN PO
784105
1975/02/06 M 44Y
2019/09/25
16:36:24
S:891:36/48
HFS
A
Henan Cancer Hospital
MR
SIEMENS Prisma
V:syngo MR E11
OP:006
A:20190921000321
R
with contrast
DIFFUSION\TRACEW\NORM\DIS2D
TR:4910 TE:56
FA:180 SAT2\SFS
Acq:1 BW:965Hz
Zoom: 1.36
THK:5.0
WW: 457 /WL: 188
