# Supplementary material for: LoG-staging: a rectal cancer staging method with LoG operator based on maximization of mutual information
Source: BMC Med Imaging. 2025 Mar 6;25:78. doi: 10.1186/s12880-025-01610-7 (PMC11887235; doi:10.1186/s12880-025-01610-7)
Supplement: Supplementary file 1 — Supplementary Material 1. [file 12880_2025_1610_MOESM1_ESM.zip › T33-eps-converted-to.pdf]

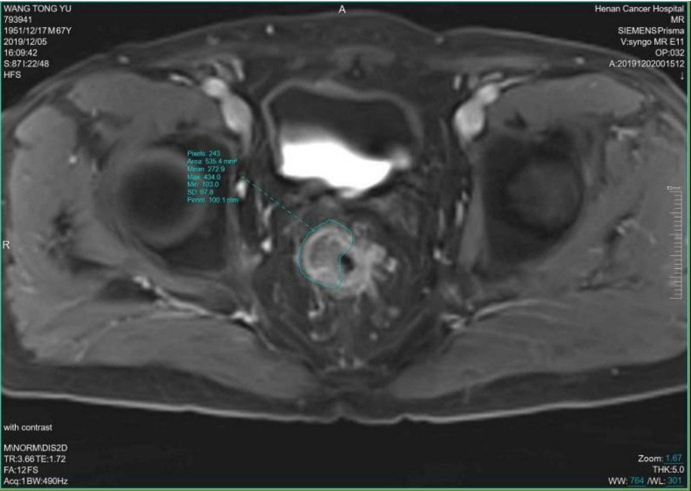
WANG TONG YU
793941
1951/12/17 M 67Y
2019/12/05
16:09:42
S:87 I:22/48
HFS
A
Henan Cancer Hospital
MR
SIEMENS Prisma
V:syngo MR E11
OP:032
A:20191202001512
Pixels: 243
Area: 535.4 mm²
Mean: 272.9
Max: 434.0
Min: 103.0
SD: 67.8
Perim: 100.1 mm
R
with contrast
M\NORM\DIS2D
TR:3.66 TE:1.72
FA:12 FS
Acq:1 BW:490Hz
Zoom: 1.67
THK:5.0
WW: 764 /WL: 301
