# Supplementary material for: LoG-staging: a rectal cancer staging method with LoG operator based on maximization of mutual information
Source: BMC Med Imaging. 2025 Mar 6;25:78. doi: 10.1186/s12880-025-01610-7 (PMC11887235; doi:10.1186/s12880-025-01610-7)
Supplement: Supplementary file 1 — Supplementary Material 1. [file 12880_2025_1610_MOESM1_ESM.zip › T41-eps-converted-to.pdf]

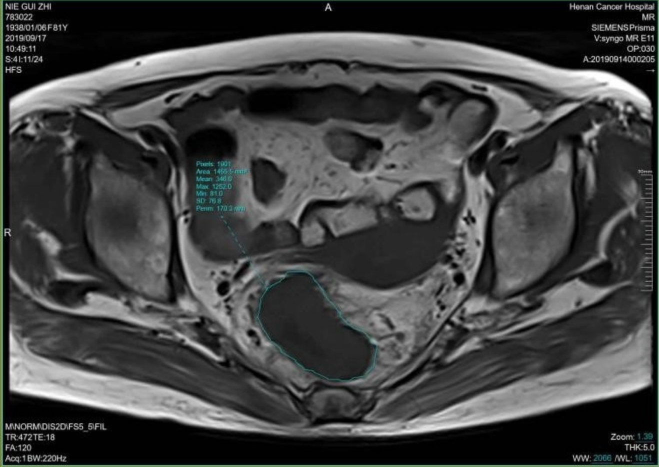
NIE GUI ZHI
783022
1938/01/06 F 81Y
2019/09/17
10:49:11
S:4l:11/24
HFS
A
Henan Cancer Hospital
MR
SIEMENS Prisma
V:syngo MR E11
OP:030
A:20190914000205
R
Pixels: 1901
Area: 1455.5 mm²
Mean: 346.6
Max: 1252.0
Min: 81.0
SD: 76.8
Perim: 170.3 mm
M\NORM\DIS2D\FS5_5\FIL
TR:472 TE:18
FA:120
Acq:1 BW:220Hz
Zoom: 1.39
THK:5.0
WW: 2066 /WL: 1051
