# Supplementary material for: LoG-staging: a rectal cancer staging method with LoG operator based on maximization of mutual information
Source: BMC Med Imaging. 2025 Mar 6;25:78. doi: 10.1186/s12880-025-01610-7 (PMC11887235; doi:10.1186/s12880-025-01610-7)
Supplement: Supplementary file 1 — Supplementary Material 1. [file 12880_2025_1610_MOESM1_ESM.zip › T42-eps-converted-to.pdf]

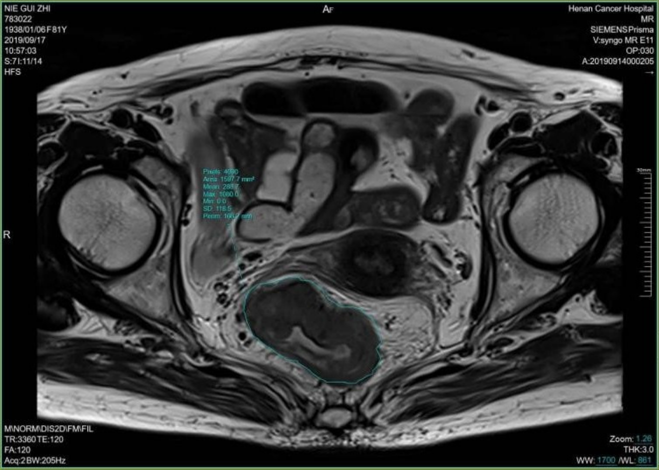
NIE GUI ZHI
783022
1938/01/06 F 81Y
2019/09/17
10:57:03
S:7I:11/14
HFS
AF
Henan Cancer Hospital
MR
SIEMENS Prisma
V:syngo MR E11
OP:030
A:20190914000205
R
Area: 1597.7 mm²
Min: 0.0
SD: 118.5
M\NORM\DIS2D\FM\FIL
TR:3360 TE:120
FA:120
Acq:2 BW:205Hz
Zoom: 1.26
THK:3.0
WW: 1700 /WL: 861
