# Supplementary material for: LoG-staging: a rectal cancer staging method with LoG operator based on maximization of mutual information
Source: BMC Med Imaging. 2025 Mar 6;25:78. doi: 10.1186/s12880-025-01610-7 (PMC11887235; doi:10.1186/s12880-025-01610-7)
Supplement: Supplementary file 1 — Supplementary Material 1. [file 12880_2025_1610_MOESM1_ESM.zip › T43-eps-converted-to.pdf]

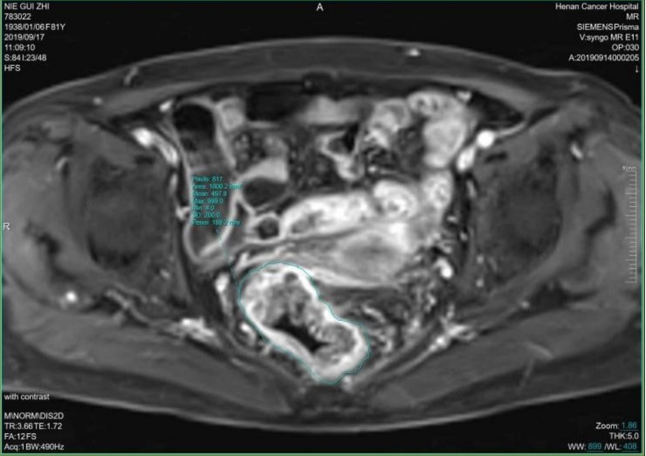
NIE GUI ZHI
783022
1938/01/06 F81Y
2019/09/17
11:09:10
S:84 I:23/48
HFS
A
Henan Cancer Hospital
MR
SIEMENS Prisma
V:syngo MR E11
OP:030
A:20190914000205
R
with contrast
M\NORM\DIS2D
TR:3.66 TE:1.72
FA:12 FS
Acq:1 BW:490Hz
Zoom: 1.86
THK:5.0
WW: 899 /WL: 408
