# Supplementary material for: LoG-staging: a rectal cancer staging method with LoG operator based on maximization of mutual information
Source: BMC Med Imaging. 2025 Mar 6;25:78. doi: 10.1186/s12880-025-01610-7 (PMC11887235; doi:10.1186/s12880-025-01610-7)
Supplement: Supplementary file 1 — Supplementary Material 1. [file 12880_2025_1610_MOESM1_ESM.zip › T44-eps-converted-to.pdf]

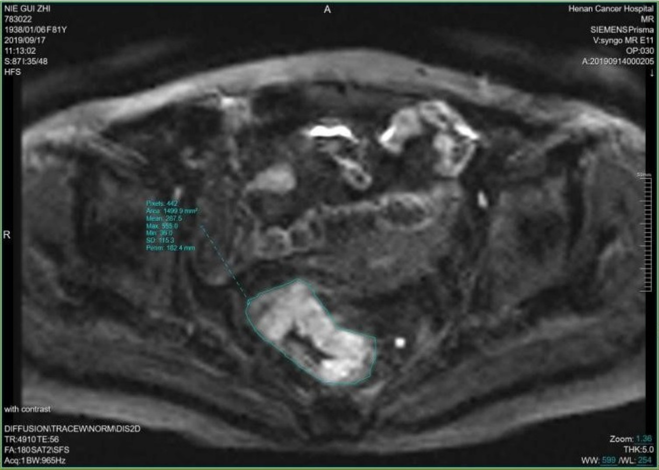
NIE GUI ZHI
783022
1938/01/06 F81Y
2019/09/17
11:13:02
S:87 I:35/48
HFS
A
Henan Cancer Hospital
MR
SIEMENS Prisma
V:syngo MR E11
OP:030
A:20190914000205
R
Pixels: 442
Area: 1499.9 mm²
Mean: 287.5
SD: 115.3
Perim: 182.4 mm
with contrast
DIFFUSION\TRACEW\NORM\DIS2D
TR:4910 TE:56
FA:180 SAT2\SFS
Acq:1 BW:965Hz
Zoom: 1.36
THK:5.0
WW: 599 /WL: 254
