# Supplementary material for: LoG-staging: a rectal cancer staging method with LoG operator based on maximization of mutual information
Source: BMC Med Imaging. 2025 Mar 6;25:78. doi: 10.1186/s12880-025-01610-7 (PMC11887235; doi:10.1186/s12880-025-01610-7)
Supplement: Supplementary file 1 — Supplementary Material 1. [file 12880_2025_1610_MOESM1_ESM.zip › T12-eps-converted-to.pdf]

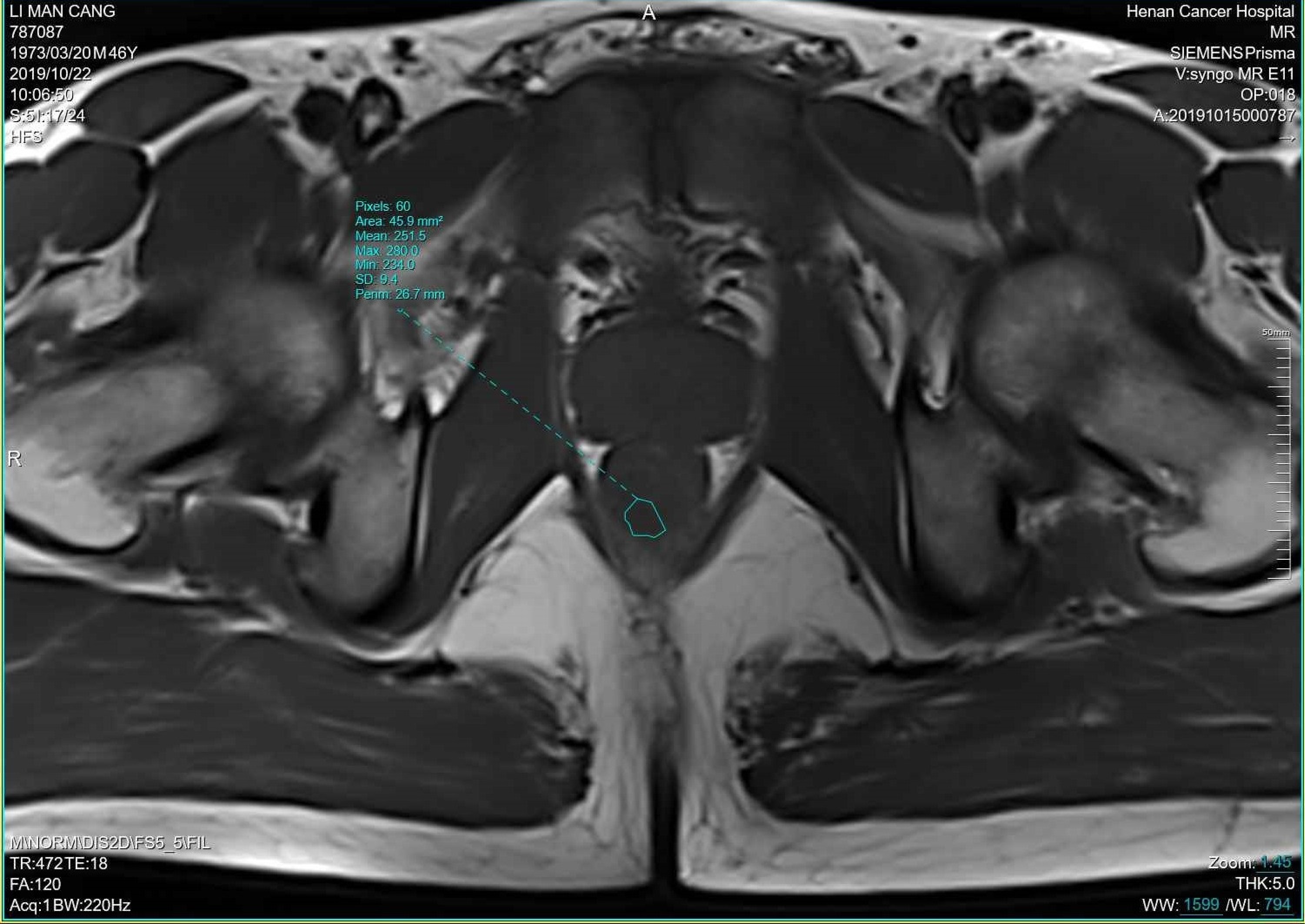

LI MAN CANG
787087
1973/03/20 M 46Y
2019/10/22
10:06:50
S:51:17/24
HFS
A
Henan Cancer Hospital
MR
SIEMENS Prisma
V:syngo MR E11
OP:018
A:20191015000787
Pixels: 60
Area: 45.9 mm²
Mean: 251.5
Max: 280.0
Min: 234.0
SD: 9.4
Perim: 26.7 mm
R
50mm
M\NORM\DIS2D\FS5_5\FIL
TR:472 TE:18
FA:120
Acq:1 BW:220Hz
Zoom: 1.45
THK:5.0
WW: 1599 /WL: 794
