# Supplementary figures and images for: LoG-staging: a rectal cancer staging method with LoG operator based on maximization of mutual information
Source: BMC Med Imaging. 2025 Mar 6;25:78. doi: 10.1186/s12880-025-01610-7 (PMC11887235; doi:10.1186/s12880-025-01610-7)

LI MAN CANG  
787087  
1973/03/20 M 46Y  
2019/10/22  
10:30:37  
S.891.28/48  
HFS

Henan Cancer Hospital  
MR  
SIEMENS Prisma  
V.syngo MR E11  
OP:018  
A:20191015000787

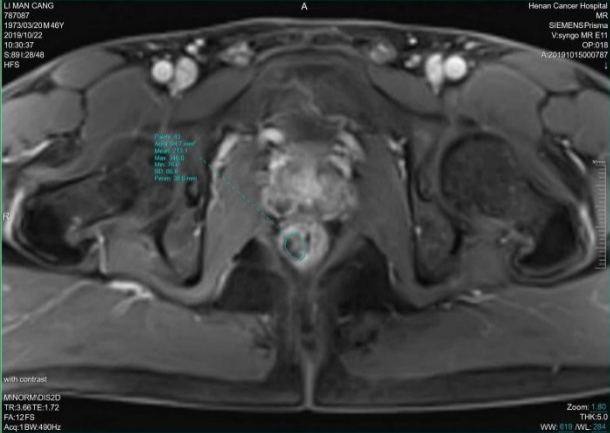

Supplement: Supplementary file 1 — Supplementary Material 1. [file 12880_2025_1610_MOESM1_ESM.zip › T13-eps-converted-to.pdf]
